# Parent Satisfaction With Care and Treatment Relates to Missed Nursing Care in Neonatal Intensive Care Units

**DOI:** 10.3389/fped.2020.00074

**Published:** 2020-03-18

**Authors:** Eileen T. Lake, Jessica G. Smith, Douglas O. Staiger, Linda A. Hatfield, Emily Cramer, Beatrice J. Kalisch, Jeannette A. Rogowski

**Affiliations:** ^1^Center for Health Outcomes and Policy Research, School of Nursing, University of Pennsylvania, Philadelphia, PA, United States; ^2^College of Nursing and Health Innovation, University of Texas at Arlington, Arlington, TX, United States; ^3^Department of Economics, Dartmouth College, Hanover, NH, United States; ^4^School of Nursing, University of Pennsylvania, Philadelphia, PA, United States; ^5^School of Nursing, University of Kansas, Kansas City, KS, United States; ^6^College of Nursing, University of Michigan, Ann Arbor, MI, United States; ^7^Department of Health Policy and Administration, The Pennsylvania State University, University Park, PA, United States

**Keywords:** parent satisfaction, missed nursing care, neonatal intensive care unit, infant, patient satisfaction, nurses, hospitals

## Abstract

**Background:** The satisfaction of parents of infants in neonatal intensive care is important to parent-infant bonding and parents' ability to care for their baby, including after discharge. Given the principal caregiver role of nurses in this setting, parent satisfaction is influenced by high quality nursing care. Nursing care that is required but missed, such as counseling and support, might influence parent satisfaction. How missed nursing care relates to parent satisfaction is unknown.

**Objective:** To describe the satisfaction of parents of infants in neonatal intensive care and to determine how satisfaction relates to missed nursing care in a sample of USA nursing units.

**Methods:** The design was cross-sectional and correlational. Thirty neonatal intensive care units that participate in the National Database of Nursing Quality Indicators were recruited. To maximize sample variation in missed care, the highest and lowest quartile hospitals on missed nursing care, measured by nurse survey, were eligible. Ten parents of infants who were to be discharged were recruited from each site to complete a survey. Parent satisfaction was measured by the EMPATHIC-38 instrument, comprising five subscales: information, care and treatment, organization, parental participation, and professional attitude, and a total satisfaction score. Multivariate regression models were estimated.

**Results:** Parent satisfaction was high (5.70 out of 6.00). The prevalence of missed care was 25 and 51% for low and high missed care units, respectively, and 40% for all units. On average, nurses missed 1.06 care activities; in the low and high missed care units the averages were 0.46 and 1.32. Over 10% of nurses missed activities that involved the parent, e.g., teaching, helping breastfeeding mothers, and preparing families for discharge. One standard deviation decrease in missed care activities at the unit level was associated with a 0.08-point increase in parent satisfaction with care and treatment (*p* = 0.01).

**Conclusion:** Parents in USA neonatal intensive care units are highly satisfied. Neonatal intensive care nurses routinely miss care. Parent satisfaction with care and treatment is related to missed nursing care. Nursing care that is missed relates primarily to the care of the baby by the parents, which could have long term health and developmental consequences.

## Introduction

Many parents of a critically ill infants are fully responsible for the infant's care upon discharge from the neonatal intensive care unit (NICU). During the infant's stay, the principal healthcare provider is the registered nurse. Effective nursing care, including communication with, and guidance and education of parents, can improve the parents' confidence and ability to care for their child at home. Therefore, communication is an integral part of family-centered care (1). When a nurse misses care, essential elements of nursing care like communication with parents does not take place. Missed nursing care occurs when required nursing care is omitted or delayed in response to multiple demands or inadequate resources (2). Missed nursing care in the NICU has not been linked to parent satisfaction. The purpose of this study was to determine the associations between missed nursing care and NICU parent satisfaction.

## Background and Significance

### Nursing and NICU Parent Satisfaction

Family-centered care in the NICU is regarded as a core strategy to improve the outcomes of critically ill infants because it acknowledges and addresses the key role of parents in the infant's health, both during inpatient care and following discharge (3). A core element of family-centered care is effective communication by nurses with parents (3). Nurse communication has been linked to NICU parent satisfaction (4–6).

When a nurse misses care related to the parent it may affect parent satisfaction. Nurses play a critical role in communicating with parents, providing education about the care of their preterm baby, and initiating breastfeeding support. Depending on the quality of nursing care of parents, including emotional support, teaching, counseling, and discharge preparation, parents may suffer emotional distress, have difficulty bonding with their infant, and be ill prepared to care for their baby after discharge.

Evidence from an integrative review of parent satisfaction with NICU care suggests that parent satisfaction with their overall NICU care experience has been high, with lower proportions of dissatisfaction across several countries, including the Unites States, the United Kingdom, Australia, Canada, South Africa, Israel, and the Netherlands (4). Communication has been found to be a consistent correlate of parent satisfaction in the NICU across parent satisfaction studies (4–6). Greater parental involvement in care is associated with better outcomes. For example, in a quasi-experimental study in a Chinese NICU, a parental educational intervention that encouraged active involvement in care for at least 4 h a day resulted in significantly better infant outcomes, including a higher breastfeeding rate and a lower readmission rate (7).

### Missed Nursing Care

Over the past decade, researchers have examined missed nursing care as an indicator of the quality of nursing care. Two systematic reviews have documented the predictors and consequences of missed nursing care in various inpatient settings (8, 9). In adult populations, missed nursing care has been linked to patient satisfaction, adverse events, mortality, and readmission (10–14). Evidence about missed nursing care in NICUs is consistent with findings from adult settings and has been growing. NICU missed care literature can be described using three major categories: system factors that related to missed care, consequences of missed care, and missed care disparities across settings.

System factors, including work environments, nurse workloads, and infant acuity, have been shown to predict missed nursing care. In a sample of 5,862 NICU staff nurses reporting about 303 USA NICUs, nurses with higher workloads, poorer work environments, and higher acuity patients had higher odds of missed care (15). In nine Quebec NICUs, missed care was less frequent in better work environments (16). Data from 230 USA certified neonatal nurses suggested that system factors, such as unit census, contribute to missed care (17). In one USA NICU, at an academic medical center, caring for three or more infants was associated with 2.5 higher odds of missed nursing care, and subjective nurse workload ratings were associated with a 34% increase in the odds of missing a care activity during a shift (18).

Consequences for infant outcomes and parent satisfaction could result from missed care in the NICU. One study in a USA medical center linked delayed feeding (a type of missed care) of NICU infants to time required to achieve full oral feedings and length of stay (19). Nurses who worked in seven Quebec NICUs reported that missed discharge teaching was inversely associated with nurse-ratings of parent-infant readiness for discharge, and that missed parental support, teaching, and infant comfort care was associated with ratings of poorer pain control (20).

Disparities among populations exposed to missed care in the NICU is also a concern. For example, data from 134 USA NICUs suggested that nurses in NICUs with a larger proportion of Black infants, compared to all other infants, were assigned more patients on average and missed almost 50% more nursing care (21).

### Study Contribution and Purpose

Missed nursing care is a modifiable characteristic of care settings. There no evidence yet on whether missed nursing care influences NICU parent satisfaction. Furthermore, multisite evidence from the USA about NICU parent satisfaction is not available. Our study, therefore, contributes by describing NICU parent satisfaction in a large USA NICU sample examining whether missed nursing care relates to variation in parent satisfaction. Multisite USA evidence will be valuable to practitioners internationally for comparison with the USA parent experience. Correlational evidence linking missed nursing care to parent satisfaction is a step toward identifying points of intervention to improve parent satisfaction. Our research may inform nurse managers about the dimensions of parent satisfaction and the relationship between missed care and parent satisfaction, so managers and staff can become aware of missed care and consider addressing it.

The purpose of this study was to describe the satisfaction of parents of infants in neonatal intensive care and to determine how satisfaction relates to missed nursing care in a sample of nursing units in the USA.

## Methods

### Design and Setting

The design was cross-sectional and correlational. The setting was the NICU.

#### Data Sources

Missed care was measured from a survey of registered nurses (RN) conducted in 2018 for the National Database of Nursing Quality Indicators® (NDNQI®), which is a voluntary program that collects data about nursing-sensitive indicators to improve patient care quality in hospitals (22). Parent satisfaction was measured from a survey of parents of NICU infants. American Hospital Association (AHA) Annual Survey data were utilized to measure hospital characteristics and the number of NICU beds. The NICU level was classified as reported to the NDNQI by the participating hospital.

#### NICU and Parent Samples

The target population included USA NICU nurses and parents. NICU inclusion criteria were having 2017 NDNQI RN survey data and falling in the top or bottom quartile on frequency of missed nursing care, as reported by staff nurse respondents and aggregated to the unit level. The top and bottom quartiles of the distribution were selected to achieve maximum variation in missed nursing care. The parent inclusion criteria were being the parent of a NICU infant being discharged to go home within the next 24 h. Exclusion criteria were if the infant died or was transferred to another NICU. An incentive of 25 US$ cash or gift card, depending on the institution's IRB requirements, was given to parents who completed the survey. In addition, because the parent survey was conducted in 2018, all NDNQI NICUs with 2018 nurse survey data, including the sample NICUs, were combined into a NDNQI 2018 NICU cohort to provide descriptive statistics for comparison with the sample that participated in the parent satisfaction study.

### Procedures

#### NICU Screening, Recruitment, and Preparation for Data Collection

We screened 271 NICUs with 2017 NDNQI NICU nurse survey data and selected 160 (80 sites were selected from the top and bottom quartiles to achieve a minimum sample of 24). The basis for selecting 160 was the expected volunteer and completion rates from previous NDNQI projects: (30% volunteer rate; 55% of volunteers complete data collection) (160 × 0.3 = 48; 48 × 0.55 = 26, which is close to but >24). After five were omitted for multiple units in the same hospital and other reasons, 155 were invited to participate. Recruitment began in February 2018 for a project start date of July 2018. Hospital incentives for participating included 250 US$ payable to the nursing unit, free continuing education for registered nurses about NICU nursing systems research, and a 1-year subscription to *American Nurse Today*.

Each NICU assigned an on-site project coordinator affiliated with the hospital to facilitate data collection. Project coordinators were responsible for recruiting parents. When a NICU infant was expected to be discharged in the next 24 h, the nurse manager contacted the project coordinator. The project coordinator approached parents about the study and performed the informed consent process. The project coordinator maintained a record of eligible and recruited parents to calculate participation rate. Project coordinators documented cases in which parents either refused to participate or were missed. Typically, a parent was missed if an infant was discharged before the project coordinator could recruit the parent. To promote consistent data collection practices across sites, webinars were held on recruitment and data collection procedures.

Institutional Review Board (IRB) approval was obtained from the University of Pennsylvania for our protocol. Each NICU obtained IRB approval through reliance on the IRB at the University of Pennsylvania, by having the project coordinator join the research team as site principal investigator or obtaining separate IRB approval through their institution. Depending on the timing of IRB approval, data collection occurred on a rolling basis from July 2018 to February 2019.

### Measures/Instruments

#### Demographic Questions

These included items about race/ethnicity, age range, level of completed education, and parent status (i.e., mother, father, both, guardian) of the respondent. Whether the infant weighed <1,500 g at birth was also included to capture very low birthweight status. Parents were asked the length of the infant's stay (in days), and on a typical day, how many hours the parent spent on the NICU.

#### EMPATHIC-38

A 38-item modified tool to measure parent satisfaction based on the original EMPowerment of PArents in THe Intensive Care 30 questionnaire (EMPATHIC-30) (23) was utilized. The EMPATHIC-30 was developed in the Netherlands to measure the satisfaction of parents of hospitalized children (23). The EMPATHIC-30 was shortened from the 65-item EMPATHIC and has demonstrated reliability and validity, while retaining essential content, and taking less time to complete (23). It encompasses the care by nurses and physicians as well as organization and cleanliness. A sample item is, “the nurses took action immediately when our child's condition worsened.” The research team, in consultation with the EMPATHIC developer (Latour), selected 6 items from the EMPATHIC-N, a 57-item parent satisfaction questionnaire which was developed for the NICU setting (24), to increase the content about the nurse's role. To increase the accuracy of item-level measurement, we separated the terms nurses and doctors into two items resulting in an addition of eight items rather than six. The eight items were included in the same domains as the EMPATHIC-N; the two instruments have consistent domains. The five EMPATHIC-38 domains are information (five items), care and treatment (12 items), organization (five items), parental participation (eight items), and professional attitude (eight items). Items were on a 1–6 Likert-type scale, with response options ranging from “certainly no” (1) to “certainly yes” (6), with a “not applicable” option. An overall score is calculated as the mean of all items.

Some of the original survey language in Dutch English was modified for USA English speakers for understandability, with Latour's approval. For our Spanish speaking USA population, we adapted a Spain Spanish version (25) to Latin American Spanish. The Latin American Spanish version was back translated by two independent reviewers who were fluent in English and Latin American Spanish until there were no discrepancies, indicating its accuracy. The psychometric properties of both adaptations were evaluated and found to be satisfactory, as will be detailed in a concurrent manuscript. Internal consistency reliability in the current sample was satisfactory, exceeding 0.70 for four of the EMPATHIC-38 domains. The fifth domain, Organization, had an alpha of 0.60. Concurrent validity was supported in significant positive correlations between the overall score and several indicators of parent satisfaction.

#### Missed Care

Missed care was measured by self-report, the most common method found in a recent systematic review that included 42 quantitative reports (26). The selected measure is a standard in the field (14, 26), comprising 12 care activities considered fundamental to the science and practice of nursing, such as patient surveillance. This set was augmented by four items designed for this study to address NICU outcomes of breastmilk feeding and hospital-acquired infection: breastfeeding support, timely feedings, hand hygiene, and central line maintenance/care. The NDNQI added “ambulation or range of motion” based on clinical rationale and consistency with similar missed care measures. The total number of missed care items was 17.

The NDNQI survey item we used to measure missed care asks, “On the most recent shift you worked, which of the following nursing activities were necessary but left undone because of time constraints?” Respondents are asked to check all that apply. Variables were created from the missed care responses: (1) a set of binary variables indicating whether any or no care was missed and whether each activity was missed and (2) a frequency variable for the number of missed nursing activities. The 30 sample units were classified as having high or low missed care based on their location in the top or bottom half of the missed care frequency distribution in 2018.

#### Data Analysis

Parent data from NICUs were linked by AHA ID to NDNQI nurse survey data and AHA hospital characteristics. Hospital and NICU characteristics for three groups were compared: the 30 recruited NICUs, all NDNQI NICUs with nurse survey data from 2018, and hospitals with a NICU from the AHA survey. Parent and infant characteristics for high missed care units, low missed care units, and all 2018 NDNQI NICUs were described. The average number of missed care items and the percent of nurses who missed at least one care activity was compared across high, low, and all 2018 NDNQI NICUs. The distribution of the prevalence of each missed care activity across all 2018 NDNQI NICUs. The correlation of the average number of care activities missed and parent satisfaction with care and treatment was calculated.

Mixed effects multi-level regression models were estimated for the effect of missed care frequency on each parent satisfaction domain and total parent satisfaction. For regression models, missed nursing care was calculated as the standardized average number of missed care activates at the unit level. Results were interpreted as the effect of a one standard deviation (SD) change in missed nursing care. Parent characteristics (i.e., Hispanic ethnicity, race, age, level of education) and an infant characteristic (i.e., low birth weight) were controlled for in all regression models. Regression model calculations were clustered at the unit level. The alpha level for significance was set at 0.05.

## Results

### Sample Characteristics

Forty NICUs volunteered (26% volunteer rate) and 30 participated (75% completion rate). The 30 NICUs comprised 15 low and 15 high missed care units from the top and bottom quartiles. The typical sample NICU was level III newborn care, and was in a non-profit, teaching hospital in the South with fewer than 500 beds (Table 1).

**Table 1:** NICU and hospital characteristics.

	**Study subsample**	**Full NDNQI sample**	**USA NICUs^**a**^**
NICU level	*N* = 30	*N* = 258	*N* = 1,027
II	23%	31%	17%
III	73%	63%	76%
IV	3%	6%	7%
	**Study subsample**	**Full NDNQI sample**	**USA hospitals with a NICU**^**b**^
Hospital number of beds	*N* = 30	*N* = 244	*N* = 908
<300	50%	56%	41%
300–499	40%	33%	33%
>500	10%	11%	26%
Ownership			
Not for profit	83%	88%	73%
For profit	17%	6%	15%
Public	0%	6%	12%
Teaching hospital status			
Academic medical center	17%	16%	0.1%
Teaching	50%	43%	81%
Non-teaching	33%	41%	19%
Geographic region			
Northeast	13%	23%	16%
Midwest	10%	24%	21%
West	10%	13%	22%
South	67%	41%	40%
NICU beds (mean)	30.3	30.9	30.1

a *American Academy of Pediatrics 2019 NICU Directory (updated Aug 13, 2019)*.

b *Source: Authors calculations from American Hospital Association 2016 Annual Hospital Survey: hospitals with non-zero neonatal intensive care beds (nicbd)*.

Although characteristics of both the full NDNQI sample and the population of USA hospitals with a NICU are presented in Table 1, we emphasize the comparison between our sample and the USA population to which we would expect to generalize our findings. Newborn care nursing units in the USA are classified into four levels, in which Level I is normal newborns and levels II through IV reflect increasing complexity of care. Our sample proportions of levels II, III, and IV were 23, 73, and 3%, quite similar to the national distribution of 17, 76, and 7%, respectively (Table 1). On most hospital characteristics, including size, teaching status, ownership, and number of NICU beds, our sample distribution was similar to that of the USA population. Our sample's hospitals were disproportionately located in the South (67 vs. 40% nationally). The average number of NICU beds in our sample was 30.3. For the NDNQI and national samples, the average number of NICU beds was about 30 (Table 1).

There were 300 parents surveyed: 10 from each NICU. Most respondents (73%) were mothers (Table 2). The typical NICU parent was non-Hispanic, White, between 25 and 34 years of age, and had at least a high school education; however, there was a range parents represented from different races, age groups, and education categories. Regarding race, most parents were White (59%), or Black or African American (21%). While about half the parents were 25–34 years old, about one fifth each were 18–24 years old or 35–44 years old. For highest level of education completed, about half had a high school degree or GED, two fifths had associate, bachelor's or higher degrees, and 8% had trade/vocational training. These characteristics did not differ across the low and high missed care unit sub-samples.

**Table 2:** Parent and infant characteristics in all 30 units and in units classified as low and high frequency of missed nursing care (*N* = 300 parents).

	**Low *N* = 150**	**High *N* = 150**	**All *N* = 300^**a**^**
**PARENT CHARACTERISTICS**			
Who completed the questionnaire			
Mother	74%	71%	73%
Father	11%	16%	13%
Mother and father together	14%	12%	13%
Other	1%	2%	1%
Hispanic or Latino(a) ethnicity			
Yes	26%	23%	24%
No	75%	77%	76%
Race			
American Indian/Alaskan native	1%	1%	1%
Asian	4%	5%	5%
Black or African American	21%	21%	21%
Native Hawaiian or other Pacific Islander	0%	0%	0%
White	59%	60%	60%
Multiracial	5%	4%	5%
Other	9%	9%	9%
Age range			
Under 17 years old	0%	1%	0.3%
18–24 years old	23%	26%	25%
25–34 years old	55%	48%	52%
35–44 years old	20%	25%	22%
45 years or older	1%	0%	1%
Highest level of education			
Grade school not completed (<8th grade completed)	1%	1%	1%
Grade school completed (8th grade completed)	3%	4%	3%
High school/ GED or equivalent	48%	37%	43%
Trade/technical/vocational training	8%	11%	10%
Associate degree	11%	17%	14%
Bachelor's degree	20%	23%	22%
Professional degree or doctorate	9%	6%	7%
Other	1%	0%	0.3%
**INFANT AND NURSING UNIT STAY CHARACTERISTICS**			
Weighed <1,500 grams at birth			
Yes	19%	20%	19%
No	81%	80%	81%
Length of stay on the nursing unit			
Days (mean)	18.4	18.9	18.7
Hours the parent spent on the nursing unit on a typical day during the infant stay			
Hours (mean)	6.7	7.6	7.1

a *The number of respondents ranged from 293 to 300 due to missing data*.

*Percentages may not add to 100 due to rounding*.

Most parents (81%) did not have a VLBW infant. The most common length of NICU stay for infants was 3 or 4 days (range = 1.5–266). Parents spent about 7 h a day on the unit, with parents in high missed care units spending one half-hour more on average than parents from low missed care units.

### Missed Care Description

**Figure 1:**
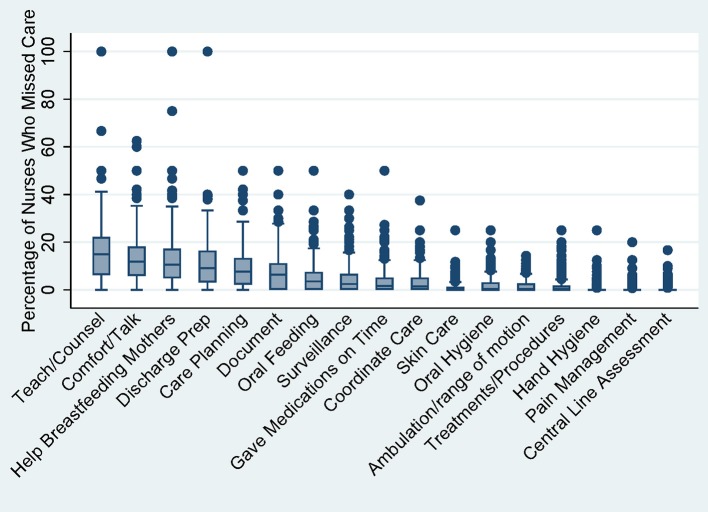
Distribution of missed care activities across 258 NIUUs.

There were 258 NDNQI NICUs with nurse survey data in 2018 for comparison with our sample. Across these 258 units, the mean percentage of nurses who missed any care was 37% and the mean frequency of missed care items was 0.95 out of 17. The median percentage of nurses who missed care (i.e., prevalence) was highest for the following four care activities: teaching and counseling patients and families (15%), comfort and talk with patients (12%), help and counsel breastfeeding mothers (11%), and prepare patients and families for discharge (9%) (Figure 1). Prevalence was lowest (0%) for oral care, skin care, ambulation, treatment and procedures, hand hygiene, pain management, and central line assessment (Figure 1).

Table 3 displays means and standard deviations for the three missed care variables in low and high missed care units compared to all 258 NICUs surveyed in 2018. The average number of missed care activities (theoretical range from 1 to 17) was 0.46 for low missed care units, 0.95 for all units, and 1.32 for high missed care units. The percentage of nurses who missed at least one care activity was 25% for low missed care units, 38% for all units, and 51% in high missed care units. The units with higher missed care activities tended, on average, to have 2–5 times higher missed care on most items except for central line assessment and care (Table 3).

**Table 3:** Distribution of missed nursing care across 2018 NDNQI nursing units: low, all, and high missed care (unit-level).

**Number of Units**	**All**	**Low**	**High**
	***N* = 258**	***N* = 15**	***N* = 15**
	**Mean (*SD*)**	**Mean (*SD*)**	**Mean (*SD*)**
Average number of missed care items in the unit	0.95 (0.64)	0.46 (0.26)	1.32 (0.31)
Percent of nurses with at least one activity missed	37.29 (18.92)	25.03 (14.70)	50.88 (13.21)
**PERCENT OF NURSES MISSING SPECIFIC CARE ACTIVITIES**			
Teach/counsel patients and family	15.50 (12.83)	7.47 (8.09)	20.57 (11.63)
Comfort/talk with patients	13.44 (10.78)	6.35 (6.66)	18.33 (8.54)
Help/counsel breastfeeding mothers	12.58 (12.13)	7.55 (7.39)	15.76 (9.40)
Prepare patients and families for discharge	10.62 (10.45)	8.76 (6.42)	11.94 (5.77)
Develop or update care plans	9.59 (9.60)	2.67 (4.98)	13.86 (10.03)
Adequately document nursing care	7.92 (7.83)	3.50 (6.59)	12.43 (8.41)
Administer oral feedings on time	5.27 (6.90)	1.54 (2.73)	7.52 (5.71)
Adequate patient surveillance	4.80 (6.68)	2.53 (4.39)	6.46 (6.96)
Administer medication on time	3.76 (5.95)	0.63 (1.95)	5.10 (4.13)
Coordinating patient care	3.38 (4.80)	0.13 (0.52)	5.73 (3.68)
Oral hygiene	2.11 (3.89)	1.25 (2.00)	4.45 (4.18)
Treatment and procedures	1.65 (3.70)	0.95 (2.17)	3.95 (6.51)
Ambulation or range of motion	1.61 (2.72)	0 (0)	1.42 (2.46)
Skin care	1.10 (2.54)	0.98 (2.66)	2.13 (3.08)
Adequate hand hygiene	0.58 (2.19)	0.73 (1.81)	1.24 (2.22)
Pain management	0.50 (1.84)	0.16 (0.63)	0.45 (1.00)
Central line assessment/care	0.61 (1.78)	0.31 (0.97)	0.18 (0.47)

*“All” refers to all NDNQI units with survey data from 2018. “Low” refers to the 15 units with missed care frequency in the bottom half of the 2018 distribution for the 30 sample units. “High” refers to the 15 units with missed care frequency in the top half of the 2018 distribution for the 30 sample units*.

### Parent Satisfaction Description

The average parent rating for total parent satisfaction was high at 5.70 (*SD* = 0.41) (theoretical range from 1 to 6). Of the five domains of the EMPATHIC-38 scale, parent satisfaction for our sample ranged from 5.61 (information domain) to 5.79 (professional attitude). There was little variation between mean scores and standard deviations for all EMPATHIC-38 subscales and the total satisfaction score (Table 4).

**Table 4:** EMPATHIC-38 descriptive statistics and multivariate regression results.

**Variable**	**M**	**SD**	**Effect of a 1 SD change in missed care**	***P*-value**
Information	5.61	0.65	−0.05	0.23
Care and treatment	5.69	0.50	−0.08	0.01
Organization	5.77	0.45	−0.01	0.65
Parental participation	5.65	0.52	0.00	0.94
Professional attitude	5.79	0.40	−0.03	0.15
Total satisfaction score	5.70	0.41	−0.04	0.09

*The number of responses from parents for EMPATHIC-38 domains and total score ranges from 299 to 300 due to missing data. Means and SDs of domains and the total score for parent satisfaction are calculated at the parent-level. For multiple regression, the independent variable is the standardized average number of missed care activities, clustered at the unit-level (30 clusters). Control variables were included in all models included parent characteristics (Hispanic or non-Hispanic, race, age, level of education) and an infant characteristic (low birthweight)*.

### Relationship Between Missed Care and Parent Satisfaction

**Figure 2:**
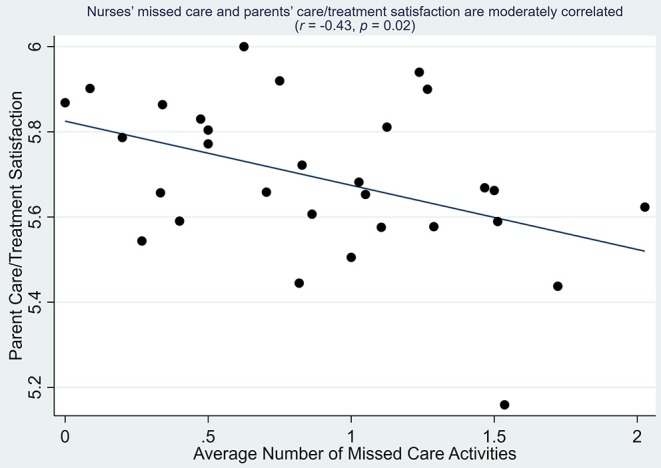
Correlation between parent satisfaction with care and treatment and average number of missed care activities (unit-level).

The average number of missed care activities was negatively correlated with the care and treatment domain of parent satisfaction (*r* = −0.43, *p* = 0.02) as it does in our Figure 2 graph. In linear regression models that controlled for parent and infant characteristics, a one standard deviation decrease in the standardized average number of missed care activities at the unit level was associated with a 0.08-point increase in parent satisfaction for the care and treatment domain of EMPATHIC-38 (*b* = −0.08, *p* = 0.01; Table 4). The association of missed care with the other domains of parent satisfaction (i.e., information, organization, parental participation, professional attitude) and the total parent satisfaction score was smaller in magnitude and not statistically significant (Table 4).

## Discussion

Based on the key role that nurses play in caring for both the infant and its parents, we theorized that parental satisfaction would relate to nursing care. So, when nurses missed care, we expected it to affect the parent's experiences. In fact, our study of 945 staff nurses and 300 parents in 30 USA NICUs showed that at units where more nursing care was missed, parents reported less satisfaction with care and treatment. To our knowledge, no other research has established a relationship between missed care and parent satisfaction, demonstrating a novel contribution of this paper.

Parent satisfaction matters because the parents' experiences during the infant's hospitalization lay the foundation for their parental role. Therefore, the significance of NICU parent satisfaction cannot be overstated. Nurses are a critical part of nurturing the parent-infant dyad. The NICU is a nurse intensive setting, where typically, one nurse cares for two infants (27). Our findings imply that the centrality of nursing to this high intensity population is important for all health care providers in this setting as well as hospital administrators to recognize. The parent/newborn dyad is unique among inpatient populations, even as compared to other intensive pediatric settings, because of this critical stage of human development. The types of care that are missed most frequently relate to the parents' role. These include teaching and counseling, comforting and talking, breastfeeding support, and discharge preparation. Breastmilk is vital to the newborn development, particularly for critically ill infants, and yet the challenges of providing breastmilk to this infant group are considerable.

The care activities missed most frequently in our study, i.e., teaching and counseling, comforting and talking, breastfeeding support, and discharge preparation, require good communication practices with the parent. Our finding that frequently missed care related to communication influences parent satisfaction is consistent with other studies that link communication and parent satisfaction in the NICU (4–6).

The care and treatment domain of the EMPATHIC-38 comprises 12 items. Two items refer to the team working closely together and attending to the prevention and treatment of pain in the child. The remaining items comprise five distinct statements asked about nurses and doctors separately, yielding ten items. These five statements refer to discharge preparation, comfort, emotional support, and acting immediately if the child's condition worsened. The high degree of congruence in content between what nurses miss most frequently and the items in the care and treatment domain indicates that parents may have awareness of missed care and it influences their satisfaction.

Parents of NICU infants in our sample were highly satisfied. This finding is consistent with NICU parent satisfaction reported across other samples globally in the NICU (24, 28) and pediatric intensive care unit (PICU) (25). For example, in a Dutch sample from which the EMPATHIC-N was derived, values ranged from 5.22 to 5.57 in a cohort of 59 parents used for confirmatory analysis (24). In that sample, the highest scores were given for Professional Attitude and the lowest scores for Information (24). Mean scores for individual items of the Italian EMPATHIC-N in an Italian sample ranged from 4.3 to 5.9 (28). In a Brazilian sample of parents, the most frequently reported satisfaction rating was between 4 and 5 for items of the EMPATHIC-N (29). In Spain, parents rated their satisfaction in the PICU as high; above 5 for most items of the EMPATHIC-30 (25).

Missed nursing care was common in our sample. Thirty-eight percent of nurses missed care on their previous shift. The average frequency of activities missed was 0.95. These statistics are similar to those reported from two earlier large samples. Compared to a sample of 2016 NDNQI NICUs with nurse survey data, the 2018 prevalence and frequency were higher: in 2016 the prevalence was 36% and the frequency was 0.88 activities (15). In a sample of 134 NICUs from four large USA states from 2006, the prevalence was 44% and the frequency was 1.23 activities (21).

Over ten percent of nurses missed activities that involve the parents, i.e., teach/counsel patients and family, comfort/talk with patients, help/counsel breastfeeding mothers, and prepare patients and families for discharge. These four activities have the most direct impact on family-centered care, as compared to physical elements of care such as skin care or treatments and procedures. These results are consistent with evidence from province-wide samples of Quebec NICUs in 2007–2008, and in 2014, which showed that the most frequently missed activities were discharge planning, parental support and teaching, and comfort care (16, 20).

In this study we measured parent satisfaction within 24 h up to expected discharge. This timing was consistent with that of previous studies, which surveyed NICU parents prior to discharge (25, 30), or on the day of discharge and up to 3 days after discharge (28). In other parent satisfaction studies, Latour et al. surveyed parents 2–3 weeks after discharge of a child from the PICU (23, 31) and 3–4 weeks after discharge of infants from the NICU (24). If satisfaction was measured later for NICU parents, e.g., 1 week after discharge, it might differ from the viewpoint at discharge. A later assessment would incorporate the parent's perspective after the parent has had time to be at home to care for the infant—and see if they felt they were adequately prepared (through nursing teaching, counseling, and education) to care for their infant at home after discharge. Future research in larger samples should be conducted to provide additional evidence about how missed nursing care relates to parent satisfaction.

### Limitations

The NICU sampling frame was NDNQI hospitals, which due to participation in a voluntary benchmarking database for quality of care, may not represent USA NICUs. The NICU sample was recruited based on high and low reported missed care values. The results do not necessarily represent all NICUs. The missed care data were unit-level aggregates and did not apply directly to the parent/infant dyads represented in our sample. As parent surveys were conducted at discharge in the hospital, parent data did not reflect satisfaction after the parents took the baby home. The geographic distribution of sample hospitals was disproportionally Southern, but all geographic regions were represented.

## Conclusion

Parents in USA neonatal intensive care units are highly satisfied at the point of discharge. Neonatal intensive care nurses routinely miss required care activities. Parent satisfaction with care and treatment is significantly related to the frequency of missed nursing care. The care activities that nurses miss relate primarily to the care of the baby by the parents once discharged, which could have long term health and developmental consequences.

## Data Availability Statement

The nurse survey data underlying the study are not available to the public as the project does not meet the National Institutes of Health minimum budget requiring data sharing. The nurse survey data are restricted due to a data use agreement with Press Ganey, Inc. The parent survey data may be available from the corresponding author. The National Database of Nursing Quality Indicators® (NDNQI®) data were supplied by Press Ganey Associates, Inc. Press Ganey Associates, Inc. specifically disclaims responsibility for any analyses, interpretations, or conclusions.

## Author Contributions

EL, JR, and DS: study concept and design. EL, EC, and JS: data acquisition. EL, JS, JR, DS, EC, and LH: data analysis and interpretation. EL, JS, DS, JR, BK, and LH: manuscript preparation. All authors reviewed and edited the final manuscript.

### Conflict of Interest

The authors declare that the research was conducted in the absence of any commercial or financial relationships that could be construed as a potential conflict of interest.
